# Supplementary material for: Geographical Distance, Socioeconomic Deprivation, and Educational Level Shape Access to Voluntary Termination of Pregnancy in a Southern Region of Italy
Source: Healthcare (Basel). 2025 Aug 29;13(17):2160. doi: 10.3390/healthcare13172160 (PMC12427977; doi:10.3390/healthcare13172160)
Supplement: Supplementary file 1 [file healthcare-13-02160-s001.zip › healthcare-3807769-supplementary.pdf]

Supplementary material

Sensitivity analyses were conducted using the Fully Conditional Specification (FCS) method to impute missing data. The results (Tables S1 and S2) confirmed the robustness of the main findings. The direction and magnitude of the associations remained consistent, with only minor variations in the estimated rate ratios (RRs) and p-values. No substantial changes were observed in the significance or interpretation of the key predictors, supporting the validity of the main analysis.

Table S1. Main characteristics of women who choose to terminate their pregnancy. Comparison between VTP practiced outside and within the catchment area in complete-case dataset and in imputed dataset.

Variable	Complete-case dataset*			Imputed dataset		
	Outside the CA n=2657	Within the CA n=2201	p	Outside the CA n=2736	Within the CA n=2261	p
Age						
< 30 years	1172 (44.1%)	972 (44.2%)	0.9711	1190 (43.5 %)	988 (43.7 %)	0.8853
≥ 30 years	1485 (55.9%)	1229 (55.8%)		1546 (56.5 %)	1273 (56.3 %)	
Educational attainment						
Diploma/degree	1558 (58.6%)	1129 (51.3%)	< 0.0001	1604 (58.6 %)	1164 (51.5 %)	< 0.0001
Other lower qualification	1099 (41.4%)	1072 (48.7%)		1132 (41.4 %)	1097 (48.5 %)	
Employment status						
Employed	1137 (42.8%)	676 (30.7%)	< 0.0001	1175 (42.9 %)	702 (31.0 %)	< 0.0001
Unemployed, Student, Houseworker, etc.	1520 (57.2%)	1525 (69.3%)		1561 (57.1 %)	1559 (69.0 %)	
Citizenship						
Italian	2436 (91.7%)	1959 (89.0%)	0.0016	2436 (91.7%)	1959 (89.0%)	0.0016
Other	221 (8.3%)	242 (11.0%)		221 (8.3%)	242 (11.0%)	
Marital status						
Married or non-marital union	930 (35.0%)	807 (36.7%)	0.2286	965 (35.3 %)	834 (36.9 %)	0.2362
Other	1727 (65.0%)	1394 (63.3%)		1771 (64.7 %)	1427 (63.1 %)	
Local health authority						
Foggia (FG)	259 (9.7%)	363 (16.5%)	<0.0001	287 (10.5 %)	399 (17.6 %)	<0.0001
Bari (BA)	1299 (48.9%)	396 (18.0%)		1326 (48.5 %)	405 (17.9 %)	
Brindisi (BR)	459 (17.3%)	231 (10.5%)		466 (17.0 %)	233 (10.3 %)	
Taranto (TA)	273 (10.3%)	190 (8.6%)		279 (10.2 %)	193 (8.5 %)	
Lecce (LE)	136 (5.1%)	712 (32.3%)		143 (5.2 %)	715 (31.6 %)	
Barletta-Andria-Trani (BT)	231 (8.7%)	309 (14.0%)		235 (8.6 %)	316 (14.0 %)	
Deprivation index of the municipality of residence						
Very Low (-2.70≤DI<-1.33)	58 (2.2%)	167 (7.6%)	< 0.0001	59 (2.2 %)	169 (7.5 %)	< 0.0001
Low (-1.33≤DI≤-0.78)	322 (12.1%)	435 (19.8%)		335 (12.2 %)	440 (19.5 %)	
Medium (-0.78≤DI≤-0.20)	700 (26.3%)	321 (14.6%)		716 (26.2 %)	325 (14.4 %)	
Hight (-0.20≤DI≤+1.52)	1577 (59.4%)	1278 (58.1%)		1626 (59.4 %)	1327 (58.7 %)	

* Data used in the main analysis

Table S2. Full coefficients of the multivariable Poisson regression model estimating the rate of mobility outside the catchment area.

A) Complete-case dataset: Data used in the main analysis

Variable		β ± SE	exp(β) [CI 95%]	p-value
N° of structures within the CA (+1)		-0.43 ± 0.07	0.65 [0.57-0.74]	<.0001
Local health authority	Bari (BA)	0.27 ± 0.07	1.31 [1.15-1.50]	0.0001
	Brindisi (BR)	0.00 ± 0.08	1.00 [0.86-1.16]	0.9842
	BAT (BT)	-0.34 ± 0.1	0.71 [0.59-0.86]	0.0003
	Foggia (FG)	-0.37 ± 0.09	0.69 [0.58-0.83]	0.0001
	Lecce (LE)	-1.49 ± 0.11	0.22 [0.18-0.28]	<.0001
	Taranto (TA) (*Ref.*)	0	1.00	
Deprivation index of the municipality of residence	Very Low	0.07 ± 0.14	1.07 [0.81-1.41]	0.6461
	Low	0.24 ± 0.07	1.28 [1.11-1.46]	0.0004
	Medium	0.18 ± 0.05	1.20 [1.09-1.33]	0.0003
	High (*Ref.*)	0	1.00	
Educational attainment	Diploma/degree	0.09 ± 0.04	1.09 [1.01-1.18]	0.0314
	Other lower qualification (*Ref.*)	0	1.00	
Employment status	Employed	0.08 ± 0.04	1.09 [1.00-1.18]	0.0448
	Unemployed, Student, Houseworker, etc. (*Ref.*)	0	1.00	
Marital status	Married or non-marital union	-0.06 ± 0.04	0.94 [0.87-1.02]	0.1288
	Other (*Ref.*)	0	1.00	
Gestational age	< 90days	-0.20 ± 0.07	0.82 [0.71-0.95]	0.0083
	≥ 90 days (*Ref.*)	0	1.00	

B) Imputed dataset.

Variable		β ± SE	exp(β) [CI 95%]	p-value
N° of structures within the CA (+1)		-0.18 ± 0.03	0.84 [0.78-0.9]	<.0001
Local health authority	Bari (BA)	0.34 ± 0.07	1.4 [1.23-1.6]	<.0001
	Brindisi (BR)	0.08 ± 0.08	1.08 [0.93-1.26]	0.2981
	BAT (BT)	-0.26 ± 0.09	0.77 [0.64-0.92]	0.0049
	Foggia (FG)	-0.3 ± 0.09	0.74 [0.62-0.88]	0.0006
	Lecce (LE)	-1.41 ± 0.11	0.24 [0.2-0.3]	<.0001
	Taranto (TA) (*Ref.*)	0	1.00	
Deprivation index of the municipality of residence	Very Low	0.09 ± 0.14	1.09 [0.83-1.44]	0.5258
	Low	0.27 ± 0.07	1.31 [1.15-1.5]	0.0001
	Medium	0.22 ± 0.05	1.24 [1.13-1.37]	<.0001
	High (*Ref.*)	0	1.00	
Educational attainment	Diploma/degree	0.09 ± 0.04	1.09 [1.01-1.18]	0.0309
	Other lower qualification (*Ref.*)	0	1.00	
Employment status	Employed	0.08 ± 0.04	1.08 [1-1.17]	0.0488
	Unemployed, Student, Houseworker, etc. (*Ref.*)	0	1.00	
Marital status	Married or non-marital union	-0.06 ± 0.04	0.94 [0.87-1.02]	0.1454
	Other (*Ref.*)	0	1.00	

Gestational age	< 90 days	-0.17 ± 0.07	0.85 [0.73-0.98]	0.0215
	≥ 90 days (*Ref.*)	0	1	

Table S3. Missingness pattern matrix.

Time certification - procedure	Facility that releases certification	Age at pregnancy	Educational attainment	Employment status	Marital status	Type of VTP	Gestational age	Deprivation index	Freq	Percentage
X	X	X	X	X	X	X	X	X	4858	97.22
X	X	X	X	X	X	X	.	X	18	0.36
X	X	X	X	X	X	.	X	X	15	0.3
X	X	X	X	X	.	X	X	X	3	0.06
X	X	X	X	.	X	X	X	X	41	0.82
X	X	X	X	.	X	X	.	X	1	0.02
X	X	X	.	X	X	X	X	X	19	0.38
X	X	X	.	X	.	X	X	X	3	0.06
X	X	X	.	.	.	X	X	X	2	0.04
.	X	X	X	X	X	X	X	.	2	0.04
.	.	X	X	X	X	X	X	X	6	0.12
.	.	X	X	X	X	X	.	X	1	0.02
.	.	X	X	.	X	X	X	X	17	0.34
.	.	X	X	.	X	X	.	X	9	0.18
.	.	X	X	.	X	.	X	X	1	0.02
.	.	X	.	.	X	X	X	X	1	0.02
